# Di-μ-bromido-bis­[benz­yl(diethyl ether)magnesium]

**DOI:** 10.1107/S1600536812025445

**Published:** 2012-06-20

**Authors:** Mark A. Nesbit, Danielle L. Gray, Gregory S. Girolami

**Affiliations:** a University of Illinois at Urbana-Champaign, School of Chemical Sciences, 600 South Mathews Avenue, Urbana, Illinois 61801, USA

## Abstract

The title benzyl Grignard reagent, [Mg_2_Br_2_(C_7_H_7_)_2_(C_4_H_10_O)_2_], was obtained by reaction of benzyl bromide with magnesium in diethyl ether, followed by crystallization from toluene. The asymmetric unit comprises one half-mol­ecule, the structural dimeric unit being generated by inversion symmetry with an Mg⋯Mg distance of 3.469 (2) Å. The Mg(II) atom exhibits a distorted tetrahedral coordination geometry. The crystal packing is defined by van der Waals inter­actions only.

## Related literature

For the structures of some other diethyl ether adducts of Grignard reagents, see: Stucky & Rundle (1964 ▶); Guggenberger & Rundle (1968 ▶); Engelhardt *et al.* (1988 ▶); Antolini *et al.* (2003 ▶); Avent *et al.* (2004 ▶). For the structures of some tetra­hydro­furan and diisopropyl ether adducts of Grignard reagents, see: Maurice (1969 ▶); Spek *et al.* (1974 ▶); Krieck *et al.* (2009 ▶).
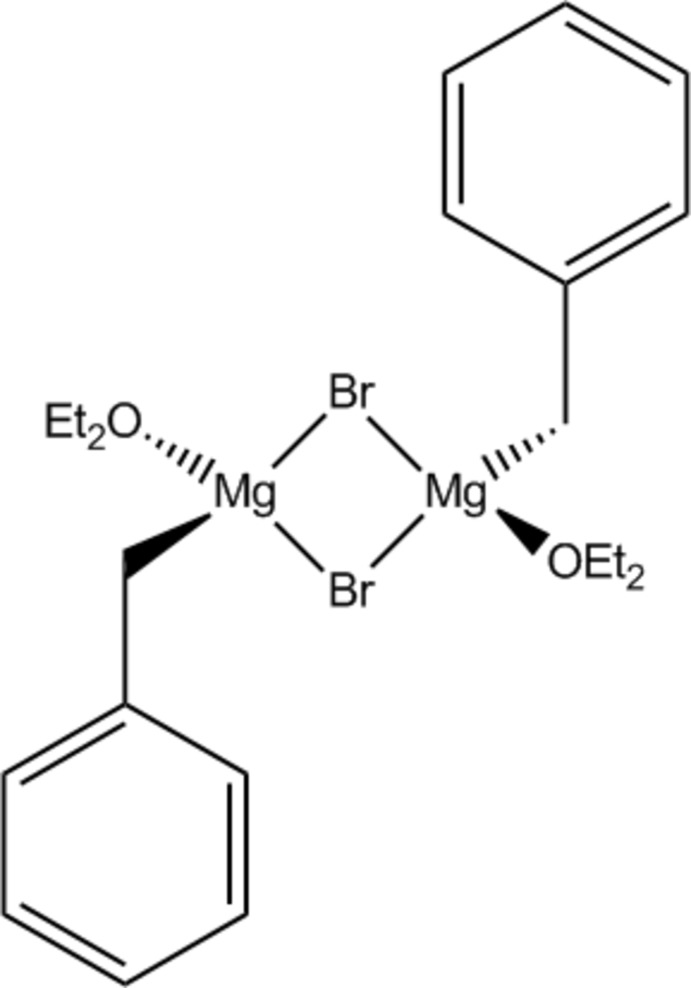


## Experimental

### 

#### Crystal data

[Mg_2_Br_2_(C_7_H_7_)_2_(C_4_H_10_O)_2_]
*M* *_r_* = 538.93
Monoclinic, 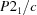

*a* = 8.0657 (4) Å
*b* = 12.4288 (6) Å
*c* = 13.1840 (6) Å
β = 96.370 (3)°
*V* = 1313.50 (11) Å^3^
*Z* = 2
Mo *K*α radiation
μ = 3.15 mm^−1^
*T* = 193 K
0.38 × 0.27 × 0.23 mm

#### Data collection

Bruker Platform APEXII CCD diffractometer
Absorption correction: integration (*SADABS*; Bruker, 2007 ▶) *T*_min_ = 0.440, *T*_max_ = 0.635
22801 measured reflections
2396 independent reflections
1808 reflections with *I* > 2σ(*I*)
*R*_int_ = 0.078

#### Refinement

*R*[*F* ^2^ > 2σ(*F* ^2^)] = 0.031
*wR*(*F* ^2^) = 0.073
*S* = 1.04
2396 reflections
129 parameters
H-atom parameters not refined
Δρ_max_ = 0.42 e Å^−3^
Δρ_min_ = −0.36 e Å^−3^

### 

Data collection: *APEX2* (Bruker, 2010 ▶); cell refinement: *SAINT* (Bruker, 2005 ▶); data reduction: *SAINT* and *XPREP* (Bruker, 2005 ▶); program(s) used to solve structure: *SHELXTL* (Sheldrick, 2008 ▶); program(s) used to refine structure: *SHELXTL*; molecular graphics: *SHELXTL* ▶; software used to prepare material for publication: *XCIF* (Bruker, 2005 ▶).

## Supplementary Material

Crystal structure: contains datablock(s) global, I. DOI: 10.1107/S1600536812025445/kp2423sup1.cif

Structure factors: contains datablock(s) I. DOI: 10.1107/S1600536812025445/kp2423Isup2.hkl

Additional supplementary materials:  crystallographic information; 3D view; checkCIF report

## Figures and Tables

**Table 1:** Selected bond lengths (Å)

Mg1—O1	2.0006 (18)
Mg1—C7	2.115 (3)
Mg1—Br1^i^	2.5448 (9)
Mg1—Br1	2.5659 (9)

Symmetry code: (i) 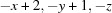.

**Fig. 1.**
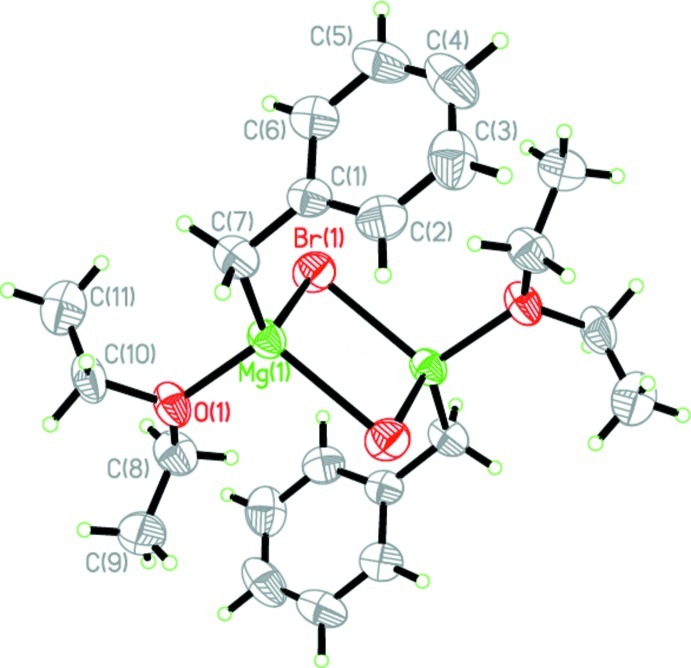
Structural unit of (I) with 50% probability displacement ellipsoids for non-H atoms. Arbitrary radii for H atoms are used. The unlabeled atoms are related by the symmetry operator (-x + 2, -y + 1, -z).

## supplementary crystallographic information

### Comment

Similar dimeric structures have been reported for the bromo Grignard reagents [Mg(µ-Br){CH(SiMe_2_Ph)(SiMe_3_)}(OEt_2_)]_2_ (Antolini, *et al.*, 2003), [Mg(µ-Br){CH(SiMe_3_)_2_}(OEt_2_)]_2_ (Avent, *et al.*, 2004), and [Mg(µ-Br)Et(O-*i*-Pr_2_)]_2_ (Spek, *et al.*, 1974). In all three of these molecules, the magnesium centres each bear one ether ligand, and two Mg–Br–Mg bridges join the metal centres. Most bromo Grignard reagents with two ether molecule per Mg centre are monomeric; examples include MgBrPh(OEt_2_)_2_ (Stucky & Rundle, 1964), MgBrEt(OEt_2_)_2_ (Guggenberger & Rundle, 1968), MgBr(CPh_3_)(OEt_2_)_2_ (Engelhardt *et al.*, 1988) and MgBr(2,4,6-C_6_H_2_Ph_3_)(thf)_2_ (Krieck *et al.*, 2009). Finally, there are some monomeric bromo Grignard reagents in which the magnesium centre bears three ether ligands and very small organic groups, such as in MgBrMe(thf)_3_ (Maurice, 1969).

### Experimental

A 250 mL round bottom flask was charged with Mg turnings (2.6 g, 107 mmol) and diethyl ether (90 mL). To the stirred suspension was added benzyl bromide (10 mL, 84 mmol) dropwise by means of an addition funnel over 30 min. After the slight exotherm had subsided, the solution was brought to reflux for two h. The solution was filtered and the filtrate was stored at 253 K. Titration with 0.13 *M* HCl showed the solution to have a concentration of 0.93 *M*.

An aliquot of the benzyl magnesium bromide solution (11.5 mL, 10.7 mmol) was taken to dryness under reduced pressure at 263 K and the residue was extracted with 1:1 benzene/toluene (50 mL). The clear yellow extract was concentrated to *ca* 20 mL and stored at 253 K overnight affording large colourless crystals.

### Refinement

A structural dimeric model of (I) is [Mg(µ-Br)(CH_2_Ph)(OEt_2_)]_2_ whereas an asymmetric unit comprises a half of the molecule. All non-H atoms were located from the difference map and refined anisotropically. H atom treatment: methyl H atom positions, R–CH_3_, were optimised by rotation about R–C bonds with idealised C–H, R–H and H–H distances; the remaining H atoms were included as riding idealised contributors. Methyl H atom *U*'s were assigned as 1.5 times *U*_eq_ of the carrier atom; remaining H atom *U*'s were assigned as 1.2 times carrier *U*_eq_.

### Crystal data

**Table:**

[Mg_2_Br_2_(C_7_H_7_)_2_(C_4_H_10_O)_2_]	*F*(000) = 552
*M**_r_* = 538.93	*D*_x_ = 1.363 Mg m^−^^3^
Monoclinic, *P*2_1_/*c*	Mo *K*α radiation, λ = 0.71073 Å
Hall symbol: -P 2ybc	Cell parameters from 5006 reflections
*a* = 8.0657 (4) Å	θ = 2.3–23.5°
*b* = 12.4288 (6) Å	µ = 3.15 mm^−^^1^
*c* = 13.1840 (6) Å	*T* = 193 K
β = 96.370 (3)°	Prism, colourless
*V* = 1313.50 (11) Å^3^	0.38 × 0.27 × 0.23 mm
*Z* = 2	

### Data collection

**Table:**

Bruker Platform APEXII CCD diffractometer	2396 independent reflections
Radiation source: normal-focus sealed tube	1808 reflections with *I* > 2σ(*I*)
Graphite monochromator	*R*_int_ = 0.078
profile data from φ and ω scans	θ_max_ = 25.3°, θ_min_ = 2.3°
Absorption correction: integration (*SADABS*; Bruker, 2007)	*h* = −9→9
*T*_min_ = 0.440, *T*_max_ = 0.635	*k* = −14→14
22801 measured reflections	*l* = −15→15

### Refinement

**Table:**

Refinement on *F*^2^	Primary atom site location: structure-invariant direct methods
Least-squares matrix: full	Secondary atom site location: difference Fourier map
*R*[*F*^2^ > 2σ(*F*^2^)] = 0.031	Hydrogen site location: inferred from neighbouring sites
*wR*(*F*^2^) = 0.073	H-atom parameters not refined
*S* = 1.04	*w* = 1/[σ^2^(*F*_o_^2^) + (0.0305*P*)^2^ + 0.0177*P*] where *P* = (*F*_o_^2^ + 2*F*_c_^2^)/3
2396 reflections	(Δ/σ)_max_ = 0.001
129 parameters	Δρ_max_ = 0.42 e Å^−^^3^
0 restraints	Δρ_min_ = −0.36 e Å^−^^3^

### Special details

**Table:**

Experimental. One distinct cell was identified using *APEX2* (Bruker, 2010). Ten frame series were integrated and filtered for statistical outliers using *SAINT* (Bruker, 2005) then corrected for absorption by integration using *SHELXTL*/*XPREP* V2005/2 (Bruker, 2005) before using *SADABS* (Bruker, 2005) to sort, merge, and scale the combined data. No decay correction was applied.
Geometry. All e.s.d.'s (except the e.s.d. in the dihedral angle between two l.s. planes) are estimated using the full covariance matrix. The cell e.s.d.'s are taken into account individually in the estimation of e.s.d.'s in distances, angles and torsion angles; correlations between e.s.d.'s in cell parameters are only used when they are defined by crystal symmetry. An approximate (isotropic) treatment of cell e.s.d.'s is used for estimating e.s.d.'s involving l.s. planes.
Refinement. The structure was phased by direct methods (Sheldrick, 2008). The systematic conditions suggested the unambiguous space group. The space group choice was confirmed by successful convergence of the full-matrix least-squares refinement on *F*^2^. The highest peaks in the final difference Fourier map were in the vicinity of atom Br1; the final map had no other significant features. A final analysis of variance between observed and calculated structure factors showed little dependence on amplitude or resolution.

### Fractional atomic coordinates and isotropic or equivalent isotropic displacement parameters (Å^2^)

**Table:**

	*x*	*y*	*z*	*U*_iso_*/*U*_eq_	
Mg1	1.18152 (11)	0.47556 (7)	0.08013 (7)	0.0396 (2)	
Br1	0.89572 (3)	0.55206 (2)	0.11052 (2)	0.05037 (13)	
O1	1.1776 (2)	0.32859 (13)	0.14171 (14)	0.0459 (5)	
C1	1.3770 (3)	0.6793 (2)	0.0998 (2)	0.0396 (7)	
C2	1.4242 (4)	0.7119 (2)	0.0058 (2)	0.0521 (8)	
H2	1.4702	0.6604	−0.0364	0.063*	
C3	1.4058 (4)	0.8173 (3)	−0.0276 (3)	0.0655 (10)	
H3	1.4394	0.8370	−0.0919	0.079*	
C4	1.3403 (4)	0.8927 (3)	0.0310 (3)	0.0712 (10)	
H4	1.3288	0.9651	0.0080	0.085*	
C5	1.2908 (4)	0.8638 (2)	0.1233 (3)	0.0624 (9)	
H5	1.2448	0.9164	0.1644	0.075*	
C6	1.3072 (3)	0.7593 (2)	0.1567 (2)	0.0488 (7)	
H6	1.2702	0.7407	0.2203	0.059*	
C7	1.3934 (3)	0.56671 (19)	0.1365 (2)	0.0468 (7)	
H7A	1.4057	0.5658	0.2120	0.056*	
H7B	1.4947	0.5340	0.1134	0.056*	
C8	1.0390 (4)	0.2534 (2)	0.1235 (2)	0.0545 (8)	
H8A	0.9597	0.2804	0.0665	0.065*	
H8B	0.9794	0.2499	0.1852	0.065*	
C9	1.0940 (4)	0.1444 (2)	0.0986 (2)	0.0708 (10)	
H9A	0.9964	0.0977	0.0838	0.106*	
H9B	1.1665	0.1153	0.1567	0.106*	
H9C	1.1557	0.1477	0.0387	0.106*	
C10	1.2862 (4)	0.3105 (2)	0.2361 (2)	0.0547 (8)	
H10A	1.3958	0.3446	0.2307	0.066*	
H10B	1.3047	0.2322	0.2457	0.066*	
C11	1.2145 (4)	0.3549 (3)	0.3267 (2)	0.0777 (10)	
H11A	1.2928	0.3431	0.3880	0.117*	
H11B	1.1088	0.3187	0.3345	0.117*	
H11C	1.1948	0.4323	0.3173	0.117*	

### Atomic displacement parameters (Å^2^)

**Table:**

	*U*^11^	*U*^22^	*U*^33^	*U*^12^	*U*^13^	*U*^23^
Mg1	0.0341 (5)	0.0340 (5)	0.0493 (6)	0.0000 (4)	−0.0015 (4)	0.0023 (4)
Br1	0.0424 (2)	0.0587 (2)	0.0502 (2)	0.00999 (14)	0.00602 (14)	−0.00680 (14)
O1	0.0356 (11)	0.0375 (10)	0.0617 (12)	−0.0040 (8)	−0.0073 (9)	0.0111 (9)
C1	0.0255 (16)	0.0405 (16)	0.0514 (18)	−0.0041 (12)	−0.0023 (13)	−0.0063 (13)
C2	0.0464 (19)	0.0541 (19)	0.056 (2)	−0.0063 (15)	0.0071 (16)	−0.0068 (15)
C3	0.059 (2)	0.069 (2)	0.069 (2)	−0.0160 (18)	0.0071 (19)	0.0179 (18)
C4	0.059 (2)	0.042 (2)	0.109 (3)	−0.0042 (17)	−0.005 (2)	0.015 (2)
C5	0.050 (2)	0.0429 (19)	0.093 (3)	0.0043 (16)	0.0063 (19)	−0.0131 (18)
C6	0.0376 (18)	0.0501 (18)	0.0593 (19)	−0.0034 (14)	0.0081 (15)	−0.0048 (14)
C7	0.0356 (17)	0.0392 (16)	0.064 (2)	−0.0012 (13)	−0.0001 (14)	0.0002 (13)
C8	0.0441 (18)	0.0441 (17)	0.074 (2)	−0.0092 (15)	0.0004 (16)	0.0044 (15)
C9	0.093 (3)	0.0498 (19)	0.068 (2)	−0.0076 (19)	0.001 (2)	−0.0049 (16)
C10	0.0472 (19)	0.0471 (18)	0.065 (2)	0.0049 (14)	−0.0134 (17)	0.0164 (15)
C11	0.087 (3)	0.085 (3)	0.060 (2)	0.004 (2)	0.002 (2)	0.0078 (19)

### Geometric parameters (Å, º)

**Table:**

Mg1—O1	2.0006 (18)	C5—C6	1.373 (4)
Mg1—C7	2.115 (3)	C5—H5	0.9500
Mg1—Br1^i^	2.5448 (9)	C6—H6	0.9500
Mg1—Br1	2.5659 (9)	C7—H7A	0.9900
Mg1—Mg1^i^	3.4690 (17)	C7—H7B	0.9900
Br1—Mg1^i^	2.5448 (9)	C8—C9	1.474 (4)
O1—C8	1.456 (3)	C8—H8A	0.9900
O1—C10	1.458 (3)	C8—H8B	0.9900
C1—C2	1.396 (4)	C9—H9A	0.9800
C1—C6	1.401 (3)	C9—H9B	0.9800
C1—C7	1.482 (3)	C9—H9C	0.9800
C2—C3	1.385 (4)	C10—C11	1.490 (4)
C2—H2	0.9500	C10—H10A	0.9900
C3—C4	1.358 (4)	C10—H10B	0.9900
C3—H3	0.9500	C11—H11A	0.9800
C4—C5	1.371 (4)	C11—H11B	0.9800
C4—H4	0.9500	C11—H11C	0.9800
			
O1—Mg1—C7	113.26 (9)	C1—C6—H6	119.0
O1—Mg1—Br1^i^	105.34 (6)	C1—C7—Mg1	110.56 (17)
C7—Mg1—Br1^i^	121.26 (9)	C1—C7—H7A	109.5
O1—Mg1—Br1	102.71 (6)	Mg1—C7—H7A	109.5
C7—Mg1—Br1	116.84 (8)	C1—C7—H7B	109.5
Br1^i^—Mg1—Br1	94.50 (3)	Mg1—C7—H7B	109.5
O1—Mg1—Mg1^i^	110.90 (6)	H7A—C7—H7B	108.1
C7—Mg1—Mg1^i^	135.62 (8)	O1—C8—C9	112.5 (3)
Br1^i^—Mg1—Mg1^i^	47.51 (2)	O1—C8—H8A	109.1
Br1—Mg1—Mg1^i^	47.00 (2)	C9—C8—H8A	109.1
Mg1^i^—Br1—Mg1	85.50 (3)	O1—C8—H8B	109.1
C8—O1—C10	114.8 (2)	C9—C8—H8B	109.1
C8—O1—Mg1	124.43 (16)	H8A—C8—H8B	107.8
C10—O1—Mg1	116.89 (15)	C8—C9—H9A	109.5
C2—C1—C6	115.8 (3)	C8—C9—H9B	109.5
C2—C1—C7	122.7 (3)	H9A—C9—H9B	109.5
C6—C1—C7	121.5 (3)	C8—C9—H9C	109.5
C3—C2—C1	121.7 (3)	H9A—C9—H9C	109.5
C3—C2—H2	119.1	H9B—C9—H9C	109.5
C1—C2—H2	119.1	O1—C10—C11	112.2 (2)
C4—C3—C2	120.5 (3)	O1—C10—H10A	109.2
C4—C3—H3	119.7	C11—C10—H10A	109.2
C2—C3—H3	119.7	O1—C10—H10B	109.2
C3—C4—C5	119.6 (3)	C11—C10—H10B	109.2
C3—C4—H4	120.2	H10A—C10—H10B	107.9
C5—C4—H4	120.2	C10—C11—H11A	109.5
C4—C5—C6	120.3 (3)	C10—C11—H11B	109.5
C4—C5—H5	119.8	H11A—C11—H11B	109.5
C6—C5—H5	119.8	C10—C11—H11C	109.5
C5—C6—C1	122.0 (3)	H11A—C11—H11C	109.5
C5—C6—H6	119.0	H11B—C11—H11C	109.5

Symmetry code: (i) −*x*+2, −*y*+1, −*z*.

## References

[bb1] Antolini, F., Hitchcock, P. B., Lappert, M. F. & Wei, X.-H. (2003). *Organometallics*, **22**, 2505—2516.

[bb2] Avent, A. G., Caro, C. F., Hitchcock, P. B., Lappert, M. F., Li, Z. & Wei, X.-H. (2004). *Dalton Trans.* pp. 1567–1577. 10.1039/b316695n 15252606

[bb3] Bruker (2005). *SAINT*, *XCIF* and *XPREP* Bruker AXS, Inc., Madison, Wisconsin, USA.

[bb4] Bruker (2007). *SADABS* Bruker AXS, Inc., Madison, Wisconsin, USA.

[bb5] Bruker (2010). *APEX2* Bruker AXS, Inc., Madison, Wisconsin, USA.

[bb6] Engelhardt, L. M., Harvey, S., Raston, C. L. & White, A. H. (1988). *J. Organomet. Chem.* **341**, 39—51.

[bb7] Guggenberger, L. J. & Rundle, R. E. (1968). *J. Am. Chem. Soc.* **90**, 5375—5378.

[bb8] Krieck, S., Görls, H., Yu, L., Reiher, M. & Westerhausen, M. (2009). *J. Am. Chem. Soc.* **131**, 2977—2985. 10.1021/ja808524y 19193100

[bb9] Maurice, V. J. (1969). *J. Organomet. Chem.* **20**, 1–10.

[bb10] Sheldrick, G. M. (2008). *Acta Cryst.* A**64**, 112–122. 10.1107/S0108767307043930 18156677

[bb11] Spek, A. L., Voorbergen, P., Schat, G., Blomberg, C. & Bickelhaupt, F. J. (1974). *J. Organomet. Chem.* **77**, 147–151.

[bb12] Stucky, G. & Rundle, R. E. (1964). *J. Am. Chem. Soc.* **86**, 4825–4830.

